# Effects of developmental stages, sex difference, and diet types of the host marmalade hoverfly (*Episyrphus balteatus)* on symbiotic bacteria

**DOI:** 10.3389/fmicb.2024.1433909

**Published:** 2024-09-04

**Authors:** Xiaoyun Wang, Ningbo Huangfu, Lulu Chen, Kaixin Zhang, Dongyang Li, Xueke Gao, Bingbing Li, Li Wang, Xiangzhen Zhu, Jichao Ji, Junyu Luo, Jinjie Cui

**Affiliations:** ^1^State Key Laboratory of Cotton Bio-breeding and Integrated Utilization, Institute of Cotton Research, Chinese Academy of Agricultural Sciences, Anyang, China; ^2^Hubei Insect Resources Utilization and Sustainable Pest Management Key Laboratory, College of Plant Science and Technology, Huazhong Agricultural University, Wuhan, Hubei, China; ^3^Xinjiang Tianyu Agricultural Science Modern Agricultural Industrialization Development Co., Ltd., Xinjiang, China; ^4^Western Agricultural Research Center, Chinese Academy of Agricultural Sciences, Changji, China; ^5^Key Laboratory of Plant Stress Biology, College of Life Sciences, Henan University, Kaifeng, Henan, China; ^6^Zhengzhou Research Base, National Key Laboratory of Cotton Bio-breeding and Integrated Utilization, School of Agricultural Sciences, Zhengzhou University, Zhengzhou, China

**Keywords:** marmalade hoverfly, symbiont dynamics, 16S rRNA sequencing, development stages, diet type, horizontal or vertical transmission

## Abstract

**Introduction:**

Symbiotic bacteria play key roles in a variety of important life processes of insects such as development, reproduction and environmental adaptation, and the elucidation of symbiont population structure and dynamics is crucial for revealing the underlying regulatory mechanisms. The marmalade hoverfly (*Episyrphus balteatus*) is not only a remarkable aphid predator, but also a worldwide pollinator second to honeybees. However, its symbiont composition and dynamics remain unclear.

**Methods:**

Herein, we investigate the symbiotic bacterial dynamics in marmalade hoverfly throughout whole life cycle, across two sexes, and in its prey *Megoura crassicauda* by 16S rRNA sequencing.

**Results:**

In general, the dominant phyla were Proteobacteria and Firmicutes, and the dominant genera were *Serratia* and *Wolbachia*. *Serratia* mainly existed in the larval stage of hoverfly with the highest relative abundance of 86.24% in the 1st instar larvae. *Wolbachia* was found in adults and eggs with the highest relative abundance of 62.80% in eggs. Significant difference in species diversity was observed between the adults feeding on pollen and larvae feeding on *M. crassicauda*, in which the dominant symbiotic bacteria were *Asaia* and *Serratia*, respectively. However, between two sexes, the symbionts exhibited high similarity in species composition. In addition, our results suggested that *E. balteatus* obtainded *Serratia* mainly through horizontal transmission by feeding on prey aphids, whereas it acquired *Wolbachia* mainly through intergeneration vertical transmission. Taken together, our study revealed the effects of development stages, diet types and genders of *E. balteatus* on symbionts, and explored transmission modes of dominant bacteria *Serratia* and *Wolbachia*.

**Discussion:**

Our findings lay a foundation for further studying the roles of symbiotic bacteria in *E. balteatus* life cycle, which will benefit for revealing the co-adaptation mechanisms of insects and symbiotic bacteria.

## 1 Introduction

A large number of symbiotic microorganisms inhabit insects, and they establish complex interactions with their host insects (Oliver and Martinez, 2014). These microorganisms mainly exist in the insects’ intestine, ovary (Vigneron et al., 2014), head, chest, abdomen (Zhang and Feng, 2018), blood cavity, cell (Shi et al., 2016), and other tissues (Albertson et al., 2013). Insects provide space for the colonization of symbiotic bacteria, and symbiotic bacteria also benefit the survival of insects. Symbionts can not only synthesize essential nutrients for host insects (Douglas, 2015), but also are closely related to insects’ digestion (Vilcinskas et al., 2020), detoxification (Lei et al., 2020), immunity (Wang et al., 2022), natural enemy defense (Attia et al., 2022), stress resistance (Lei et al., 2020; Raza et al., 2020), reproduction (He et al., 2022), metabolism, and other biological processes (An et al., 2019).

During the growth and development of insects, the composition of symbiotic bacteria in the body often changes dynamically. In this change, the host will lose, recombine or obtain new symbiotic bacteria, and this drastic change is most obvious in holometabolous insects. For example, in the butterfly *Heliconius erato*, *Acinetobacter* is present in larvae, but it almost disappears after metamorphosis. *Orbus* is very rare in larvae, undetectable in pupae, but abundant in newly emerging adults (Hammer et al., 2014). In the process of metamorphosis, the useful symbiotic bacteria will be retained and play different roles to maintain the overall operation. For example, some symbiotic bacteria can protect eggs (Flórez et al., 2017) and overwintering larva (Kaltenpoth et al., 2005), and some can digest food eaten (Gibson and Hunter, 2005; Weiss and Kaltenpoth, 2016) or provide essential nutrients at different developmental stage (Vigneron et al., 2014; Paludo et al., 2018). The symbiotic bacteria in *Camponotus* and *Cardiocondyla* ants are beneficial to the development of pupae and adults, and their metabolites provide nutrients for pupae and promote adult epidermal synthesis. In addition, the host will actively avoid the disappearance of these symbionts, so the relative abundance of these symbionts will increase during development (Wolschin et al., 2004; Klein et al., 2016). During development, some unnecessary and ineffective symbiotic bacteria will disappear after development to the next stage. At the same time, studies have also found that symbiotic bacteria can provide defense for the pupal stage of insects. For example, *Enterococcu*s in *Galleria mellonella* can produce antimicrobial peptides and inhibit the invasion of pathogens in the pupal stage (Jarosz, 1979; Johnston and Rolff, 2015). Some symbionts also promote the development of insects, and some fungal symbionts in the spineless bees produce ergosterol, which promotes larval pupation (Paludo et al., 2018).

Food type is also a major factor affecting the composition of insect symbionts. Insects, like other animals, also lack the pathway for the synthesis of certain important amino acids and vitamins, and can only get adequate nutrition by ingesting food or cooperating with microorganisms (Feldhaar et al., 2007; Hosokawa et al., 2010; Bell-Roberts et al., 2019). The changes of food types and symbiotic bacteria are inseparable. Studies have shown that the composition of symbiotic bacteria in *H. axyridis* varies depending on the type of prey or diet (Huang et al., 2022). At the same time, some symbiotic bacteria can also be transmitted between predators and preys. Luo et al. found that the relative abundance of *Serratia* in *Adelphocoris suturalis* was positively correlated with the proportion of aphids in its diet, and *Serratia marcescens* was significantly accumulated in carnivorous bugs and further lead to the insects death (Luo et al., 2021). Insects gut microbes not only play a role in providing nutrients (Shamjana et al., 2024), they can also help insects digest and contribute to the overall construction of insects (Rajagopal, 2009). Some insects that feed on plants usually contain bacteria that can degrade cell walls, which are used to degrade cellulose and pectin in cell walls (Salem et al., 2017; Gales et al., 2018).

The dynamic change between host and microorganism is not only influenced by development and food, but also by gender. This factor has been well studied in several insects. For example, it was found that there was a significant difference in midgut bacterial community composition between male and female of *Pectinophora gossypiella*, and the species richness of intestinal bacterial community was significantly higher in males than that of females (Chaitra et al., 2022). Similar reports were also found in stag beetles *Odontolabis fallaciosa* (Wan et al., 2020). While insect genders can affect the composition of symbionts, symbionts also can affect physiology and behavior of insects among two sexes. For example, symbiotic bacteria were reported that can affect the food selection of male and female insects (Schmid et al., 2014). Besides, some special symbiotic bacteria such as *Wolbachia* can also induce the feminization of insects, male-killing, and then affect the reproduction of insects eventually (Werren et al., 2008).

*Episyrphus balteatus*, also well known as the marmalade hoverfly, is a complete metamorphosis insect, which includes four developmental across the life cycle, namely eggs, larvae, pupae, and adults. Its larvae feed on aphids while adults feed on pollen nectar. As one important migratory hoverfly species, it can migrate over long distance seasonally, transporting billions of pollens and consuming trillions of aphids (Wotton et al., 2019; Clem et al., 2023). This hoverfly species is one of the most widespread species of hoverfly in the world, ranging from Asia and Europe to Africa and Australia, especially dominating in China (Yang et al., 2002). Considering the declining number of global pollinators, especially bees (Biesmeijer et al., 2006; Potts et al., 2010), hoverflies including *E. balteatus* become increasingly important (Yuan et al., 2022). However, there is limited knowledge about symbionts in hoverflies.

In this study, *E. balteatus* was selected as representative model of hoverflies species, and the microbiota communities and their dynamics across different developmental stages and genders of *E. balteatus* were investigated via 16S rRNA sequencing technology. In addition, microbiomes of hoverfly’s prey aphid *M. crassicauda* were also investigated, and the subsequently symbiotic genera shared by *E. balteatus* and *M. crassicauda* were identified to explore the possibility of symbiont horizontal transmission from prey *M. crassicauda* to marmalade hoverfly. In summary, this study revealed the changes of microbial community diversity in different developmental stages of *E. balteatus*, elucidated the acquisition (pollen and aphid) and transmission (horizontal and vertical) pathways of these microorganisms. The results showed that food types and developmental stages, were main factors influencing the dynamic changes of symbiotic microorganisms in *E. balteatus*. These findings deepen our understanding of the adaptation of insect symbionts to their host development and food changes, and provide a theoretical basis for investigating the co-adaptation of insects and microorganisms.

## 2 Materials and methods

### 2.1 Insect rearing and maintenance

The insects used in this study were all originally collected from Anyang fields (Henan Province, China, 36°5′34.8″ N, 114°31′47.19″ E). Both *E. balteatus* and *M. crassicauda* were cultured for multiple generations in climate chambers at 26 ± 1°C with a relative humidity of 75 ± 5% and a photoperiod of 16 h light: 8 h dark. *E. balteatus* adults were fed with pollens and 10% honey water, while larvae were fed with *M. crassicauda* aphids which were raised on *Vicia faba* seedlings.

### 2.2 Sampling, DNA extraction, and 16S rRNA sequencing

The eggs, the 1st instar larvae (L1), the 2nd (L2), the 3rd (L3), pupae, three-day-old female (FA) and male (MA) *E. balteatus* adults, and *M. crassicauda* individuals pooled from all development stages (1st to 4th instar nymphs and adult), were collected, respectively, and soaked in 75% ethanol solution for 30 s, separately, then washed with ultrapure water for three times. There were four biological replicates for the samples at each development stage, with each sample containing >200 eggs, 50 1st instar larvae, 30 2nd instar larvae, 20 3rd instar larvae, 20 pupae, 20 female or 20 male adults for *E. balteatus*, and 50 individuals for *M. crassicauda*, respectively. Total DNA was extracted by using a Fast DNA Spin Kit for Soil (MP, United States) according to the manufacturers’ instructions. The quantity and quality of obtained DNA were examined by Nanodrop 2000C microspectrophotometer (Thermo Fisher Scientific, United States) and 1% gel electrophoresis, respectively.

The hypervariable regions V3-V4 of bacterial 16S rRNA genes were amplified with primer pair 338F (5’-ACTCCTACGGGAGGCAGCAG-3′) and 806R(5’-GGACTACHVGGGTWTCTAAT-3′) (Liu et al., 2016) by an ABI GeneAmp^®^ 9,700 PCR thermocycler (ABI, USA). The 20 μL PCR reaction system contained 4 μL 5 × Fast Pfu buffer, 2 μL 2.5 mM dNTPs, 0.8 μL each primer (5 μM), 0.4 μL Fast Pfu polymerase, 10 ng of template DNA, and additional ddH_2_O. PCR conditions were as follows: pre-denaturation at 95°C for 3 min, followed by 30 cycles of denaturing at 95°C for 30 s, annealing at 55°C for 30 s, and extension at 72°C for 30 s, final extension at 72°C for 10 min, and ending at 4°C. The obtained PCR products were purified using the AxyPrep DNA Gel Extraction Kit (Axygen Biosciences, USA), examined by 1% agarose gel electrophoresis, and quantified using the Quantus TM Fluorometer (Promega, USA). The PCR products of the same sample were subjected to gel recovery, and purified with AmpureXP magnetic beads, and eluted with elution buffer.

### 2.3 Illumina sequencing

The symbionts in the samples were sequenced on the Illumina Mi Seq PE 300 platform (Shanghai Meiji Biomedical Technology Co., Ltd.). First, the raw reads of Illumina sequencing were filtered using fastp (Chen S. F. et al., 2018) software. Then, the results were spliced using FLASH software according to previously reported method (Magoc and Salzberg, 2011). Using UPARSE software (Edgar, 2013), the sequences obtained after quality control splicing were clustered by operational taxonomic units (OTUs), and chimeras were removed according to 97% similarity. The sequences were aligned against the Silva16S rRNA gene database (v138) using the RDP classifier (Wang et al., 2007) for OTU annotation. The confidence threshold was set as 70%, and the community composition of each sample was examined at different taxonomic levels. The original sequence obtained in this experiment was stored in the GenBank SRA database, and the accession number was PRJNA1109813. We employed the Kruskal-Wallis method to investigate the α-diversity between groups, and the false discovery rate (FDR) was used to correct the multiple tests. The statistically significant difference in symbiont abundance between groups was analyzed. *p* < 0.05 was considered as statistically significant. PICRUSt2 (Douglas et al., 2020) software was used to predict the function of symbionts, and the abundance of each functional category was calculated according to KEGG database and the abundance information of OTUs.

### 2.4 Phylogenetic analysis of *Serratia* and *Enterococcus*

To explore the evolutionary relationship between *Serratia* and *Enterococcus*, we searched the DNA sequences of these two symbiotic bacteria from NCBI database. Twenty 16S rRNA fragments were downloaded from GenBank, and the phylogenetic tree was constructed using MEGA 6.0 software by the neighbor-joining method (1,000 bootstraps).

### 2.5 Quantification of symbionts

The quantitative polymerase chain reaction (qPCR) was used to determine the 16S rRNA gene copy number of dominant symbiotic bacteria in the samples of *E. balteatus* and *M. crassicauda,* according to previously established method (Zhang et al., 2019). Specifically, the DNA of *E. balteatus* was diluted 10 times as a template for PCR amplification. The specific primers targeting dominant symbionts (Supplementary Table S1) were amplified by PCR to obtain the target sequences. The target sequences were spliced to the plasmid pEASY-T3 (Transgenic Biotechnology, China) to obtain a recombined plasmid, and the resultant plasmid was sequenced to ensure correctness of sequence. Then the recombined plasmid was subjected to five-fold gradient dilution to generate a standard curve. The qPCR was performed on Step OnePlus™ Real-Time PCR System (Applied Biosystems, United States). The 10 μL reaction system contained 5 μL 2 × TransStart Green qPCR SuperMix (TransGen Biotech, China), 0.4 μL separate forward and reverse primers, 2 μL DNA template, 2.2 μL DNase-free water. The PCR procedure was as follows: 94°C for 30s, followed by 40 cycles of 94°C for 5 s and 60°C for 30 s.

## 3 Results

### 3.1 Overview of *E. balteatus* microbiota

The microbial communities of *E. balteatus* and *M. crassicauda* were analyzed by 16S rRNA sequencing. A total of 1,391,214 clean reads and 1,142 OTUs were obtained, with an average sequence length of 422 bp (Supplementary Table S2). The dilution curve (Supplementary Figure S1) of all samples tended to be saturated after the sequence number of all sample reached 30,000, indicating most of the symbiotic microorganisms in all samples were captured. In addition, the average sequencing coverage (Good’s coverage) of each sample exceeded 99.9% (Supplementary Table S2), which further confirmed that most of the symbiotic communities in all samples were captured.

**Figure 1:**
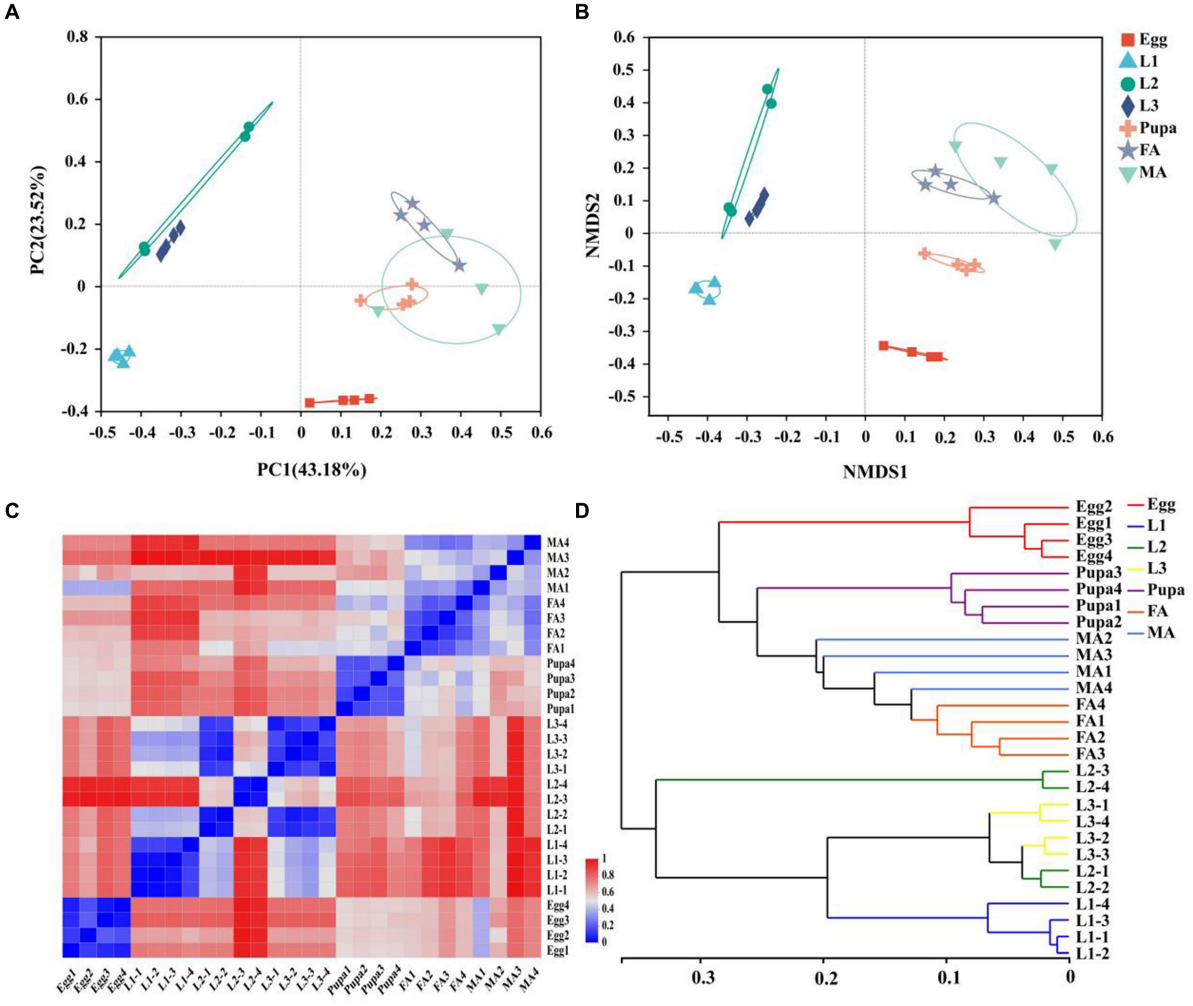
Relationship between samples at different developmental stages. **(A,B)** PCoA **(A)** and NMDS **(B)** of β diversity of symbionts in marmalade hoverfly at different developmental stages based on the Bray-Curtis distance at the OTU level. **(C)** Hierarchical clustering heatmap of symbionts at the genus level in 28 *E. balteatus* samples at different developmental stages. 0 represents the nearest distance between samples, and 1 represents the farthest distance between samples. **(D)** Hierarchical clustering tree of symbionts at the genus level in 28 *E. balteatus* samples at different developmental stages. The sample distance in the same branch was 0.35, and 0 indicates the same composition, and 1 indicates that different composition. Egg, L1, L2, L3, Pupa, FA, MA represent egg, 1st instar to 3rd instar nymphs, pupa, female and male adults of *E. balteatus*, respectively.

Both the principal coordinate analysis (PCoA) and nonmetric multidimensional scaling (NMDS) showed that symbionts of *E. balteatus* across different developmental stages and two sexes exhibited obvious intergroup dispersion and intragroup aggregation. Specifically, the groups of female adults, male adults, and pupae were closer to each other, showing high similarity among them. Meanwhile, samples of the 2nd instar and 3rd instar larvae were clustered mutually, indicating high similarity in the microbiota community between these two developmental stages (Figures 1A,B).

The heatmap of the sample distance matrix at genus level showed that the samples were divided into six modules, with the samples from egg, 1st instar larva, 2nd instar larva, 3rd instar larva, pupa, and adult (female and male) clustered respectively, indicating the variation of microbiota across different development stages of hoverfly (Figure 1C). Furthermore, hierarchical clustering analysis revealed that all samples divided into two branches, namely, the “adult & pupa &egg” branch and the larva branch (Figure 1D). The first branch was composed of pupae, adults (female and male), and eggs, and the second branch was composed of the 1st instar, 2nd instar, and 3rd instar larvae. These results suggested that the symbiont community within one branch was highly similar, whereas the symbiont community between the two branches was lowly similar.

### 3.2 Dynamics of microbiota community in *E. balteatus* across different developmental stages

**Figure 2:**
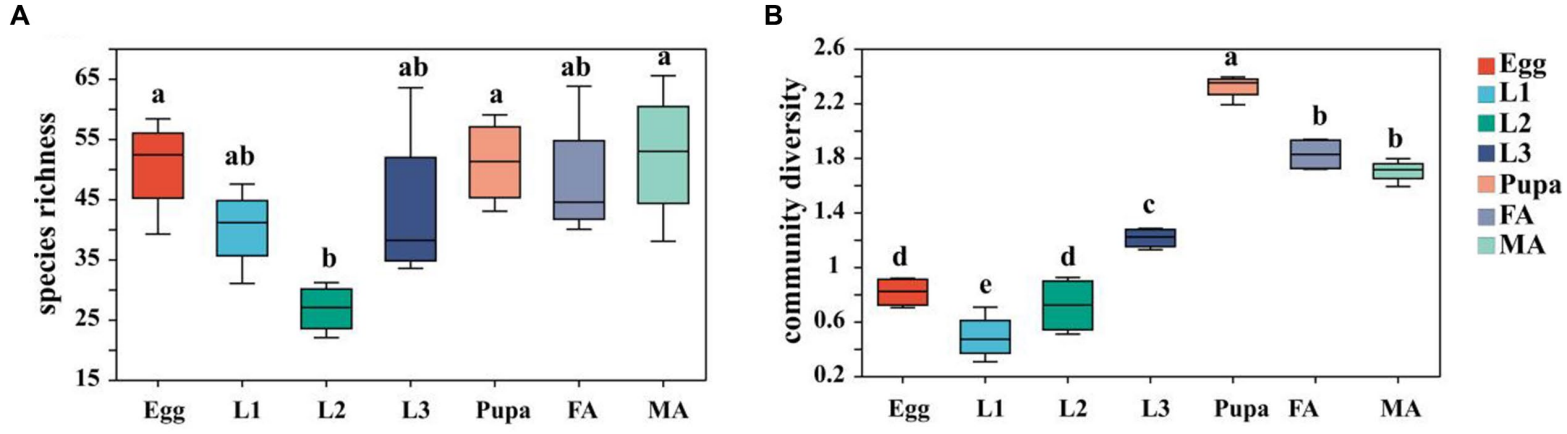
Box plot of α-diversity index of microbiota in *E. balteatus* at different developmental stages. **(A)** Chao1 index representing species richness. **(B)** Shannon index representing the community diversity of species. Egg, L1, L2, L3, Pupa, FA, MA represent egg, 1st instar to 3rd instar nymphs, pupa, female and male adults of *E. balteatus*, respectively.

The α-diversity index analysis results showed that the species richness of microbiota exhibited the trend of first decrease and then increase, with the development of hoverfly, plunging into the lowest point in the 2nd instar larval stage (Figure 2A; Supplementary Table S2). On the contrary, the microbiota diversity showed a trend of first increase (peaking at the pupal stage) and then slight decrease (Figure 2B; Supplementary Table S2).

**Figure 3:**
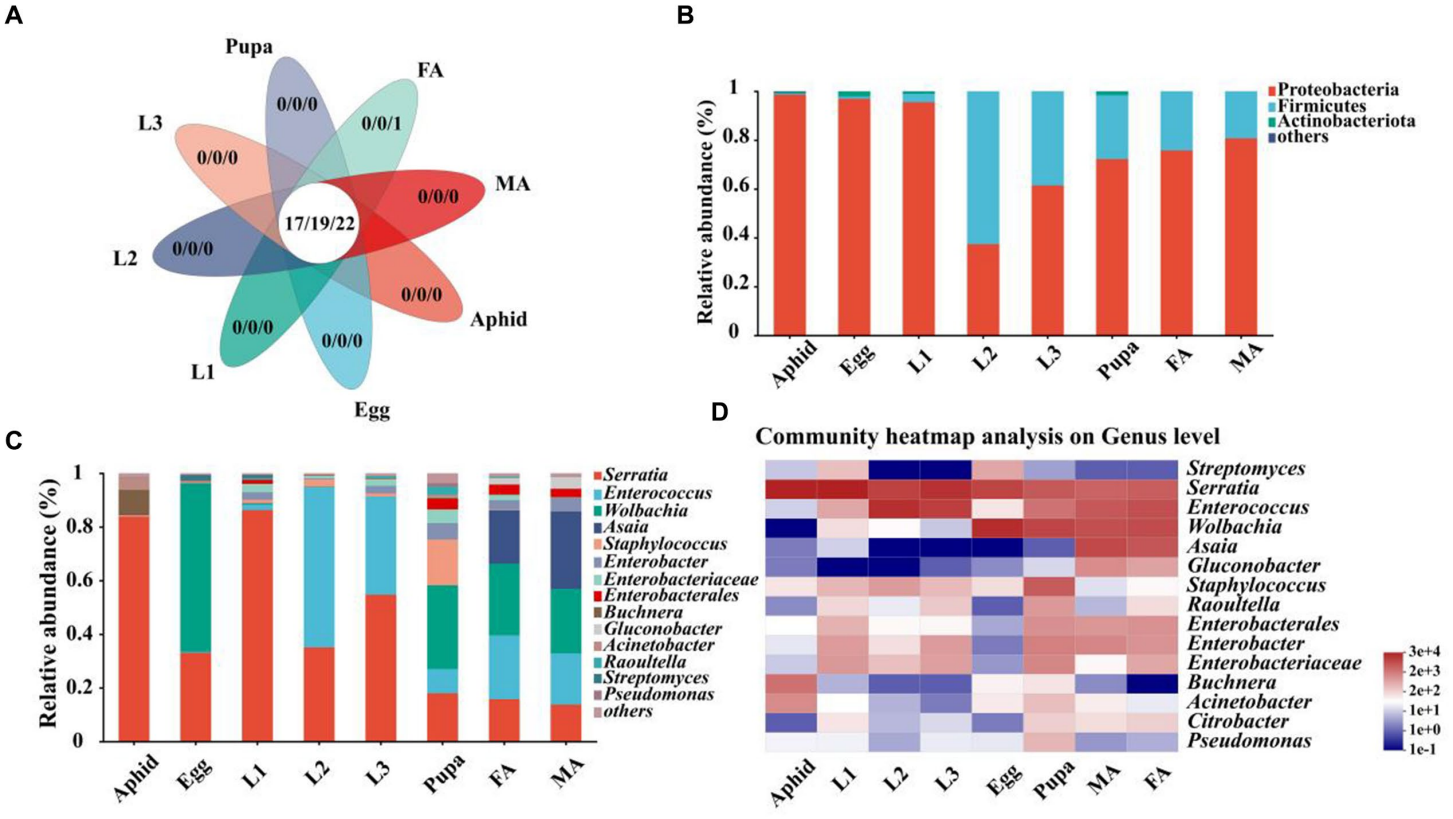
Relative abundance of symbionts in aphids and *E. balteatus* across different developmental stages. **(A)** Number of shared and unique symbionts of *E. balteatus* and *M. crassicauda* at different taxonomic levels (17 families, 19 genera, and 22 species). **(B,C)** Relative abundance of symbionts in *E. balteatus* across the development at the phylum level **(B)** and genus level **(C)**. **(D)** Heatmap of top 15 dominant symbionts of *E. balteatus* across the development at the genus level. The color gradients of different color blocks represent the richness changes of different species in the sample, and the right side of the figure is the value represented by the color gradient. Egg, L1, L2, L3, Pupa, FA, MA represent egg, 1st instar to 3rd instar nymphs, pupa, female and male adults of *E. balteatus*, respectively.

First of all, we identified multiple shared symbiotic bacteria of aphids and the hoverfly, which belonged to 17 families, 19 genera, and 22 species, respectively (Figure 3A; Supplementary Table S3). At the phylum level, Proteobacteria, Firmicutes, and Actinobacteriota were the dominant symbionts in *E. balteatus* during the development (Figure 3B; Supplementary Table S3). Specifically, Proteobacteria was the main dominant symbiont phylum, with the highest relative abundance in eggs (96.91%), and 1st instar larvae (95.49%), the lowest relative abundance in 2nd instar larvae (37.36%), respectively. Firmicutes was the dominant phylum in the 2nd instar larvae of hoverfly with a relative of 62.62% (Figure 3B; Supplementary Table S3). Besides, the symbiotic community composition of pupae and adults (female and male) was similar.

Symbiotic community of *E. balteatus* across development stages was further analyzed at the genus level. The results showed that microbiota compositions of *E. balteatus* in each developmental stage at the genus level (Figure 3C) were highly similar to those at the family level (Supplementary Figure S2). *Serratia*, *Enterococcus*, *Wolbachia*, and *Asaia* were the four preponderant genera with the highest relative abundance in all samples, and their relative abundances were significantly different between aphids and hoverfly samples (Supplementary Table S3). *Serratia* was one of the dominant symbiotic genera with the highest relative abundance in 1st instar larvae (86.24%) and 3rd instar larvae (54.69%) (Figure 3C; Supplementary Table S3). Specifically, the relative abundance of *Serratia* in aphids and hoverfly larvae (1st instar, 2nd instar, and 3rd instar) was significantly higher than in hoverfly adults (female and male). Besides, *Wolbachia* was mainly found in eggs, pupae, female adults, and male adults of hoverfly, with the highest relative abundance in eggs (62.80%). We also found that *Serratia* also occupied a high proportion in the symbiotic community composition of aphids with a relative abundance of 83.77%.

To further investigate the dominant symbiont dynamics of hoverfly across development stages, the top 15 symbiotic genera in terms of relative abundance were selected and visualized via heatmap (Figure 3D; Supplementary Table S3). *Serratia* existed in all hoverfly development stages with a high relative abundance ranging from 86.25 to 13.72% (Figure 3D; Supplementary Table S3). *Enterococcus* was another dominant bacterial genus with its relative abundance rising from egg stage (0.36%) to the 2nd instar larval stage (59.66%), followed by a gradual decrease from the 3rd instar larval stage (36.53%) to adult stage (23.81% in female and 19.10% in male). The relative abundance of *Wolbachia* in eggs, pupae, and adults (female and male) was 24.08% ~ 62.81%, which was obviously higher than that in the three larval stages. Both *Asaia* and *Gluconobacter* dominated only in female and male adults, and they were not dominant in other stages (Figure 3D; Supplementary Table S3).

### 3.3 Potential horizontal transmission of symbionts from prey *M. crassicauda* to predator *E. balteatus*

**Figure 4:**
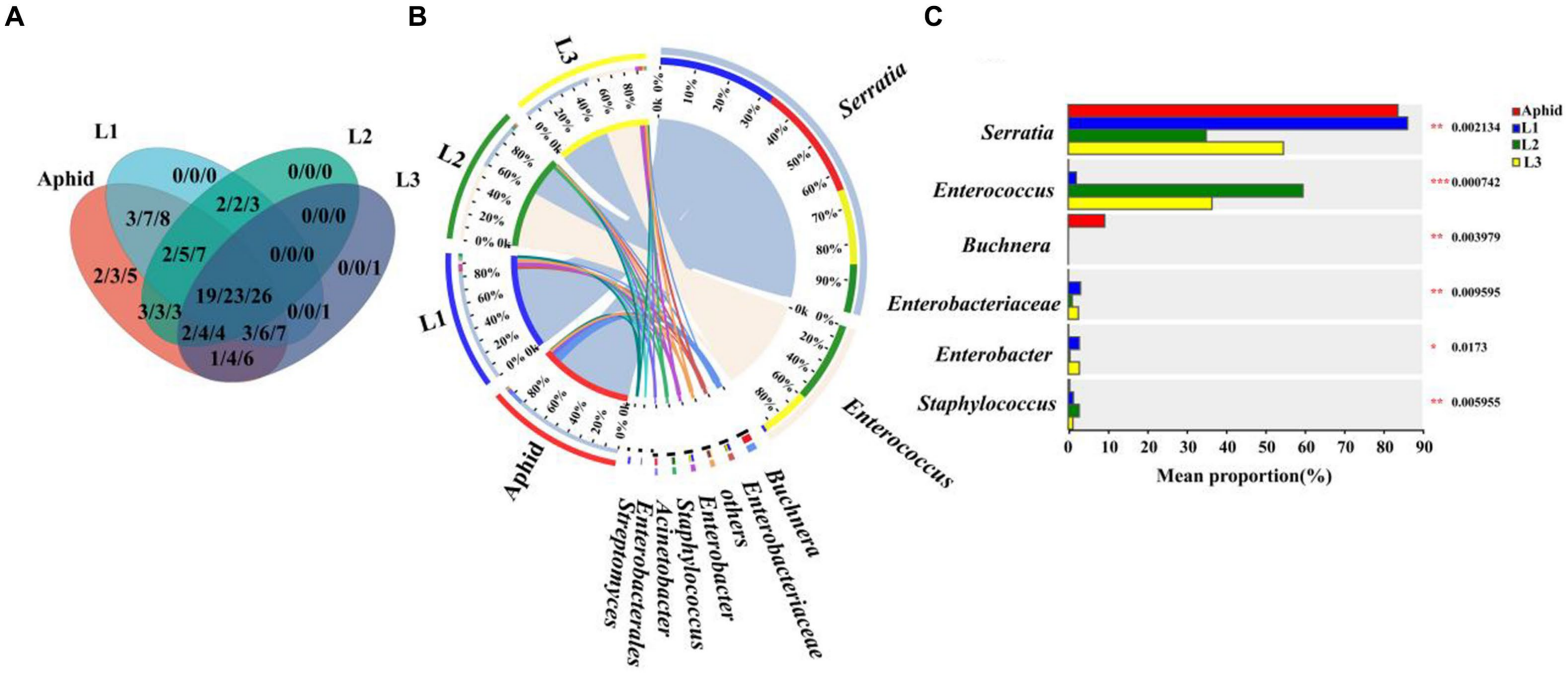
Comparison of symbiont community between *E. balteatus* larvae and *M. crassicauda*. **(A)** Shared and unique symbionts between the *E. balteatus* larvae and *M. crassicauda* at the family, genus, and species levels. Those three numbers separated by slashes represent shared or unique symbiont number at family, genus, and species level (from left to right), respectively. **(B)** Composition of symbionts at the genus level in *E. balteatus* larvae and *M. crassicauda*. **(C)** Top six symbionts with significant differences in relative abundance between *E. balteatus* larvae and *M. crassicauda* at the genus level. L1, L2, L3 represent 1st instar to 3rd instar nymphs of *E. balteatus*, respectively.

To further explore the potential horizontal transmission of symbionts from aphids to hoverflies through the food chain, the microbiota of aphids and hoverfly larvae (1st instar to 3rd instar) were analyzed at the family, genus, and species levels, respectively. Multiple symbionts shared by aphids and hoverfly larvae were obtained, belonging to 19 families, 23 genera, and 26 species, respectively (Figure 4A; Supplementary Table S4).

At the genus level, *Serratia* is the most dominant symbionts, exhibiting extremely high relative abundance in the 1st instar larvae of hoverfly and aphids, which was 84.24 and 83.77%, respectively. Its relative abundance was sharply reduced in 2nd instar and 3rd instar larvae of hoverfly, which was 35.06 and 54.69%, respectively (Figures 3C, 4B; Supplementary Table S4). These results indicated that *Serratia* was the main dominant symbiont in aphids, and that it might enter the larval body of *E. balteatus* through food chain, then gradually colonize, and become the main dominant symbiont with the development of predator. *Buchnera*, usually as an obligate symbiont in most aphid species, had a relatively low relative abundance (9.27%) in *M. crassicauda* (Figure 4C), but it was not found in marmalade hoverfly larvae, suggesting that unlike *Serratia*, *Buchnera* could not be horizontally transmitted from aphids to hoverfly.

### 3.4 Potential vertical transmission of symbionts from parent *E. balteatus* to egg

**Figure 5:**
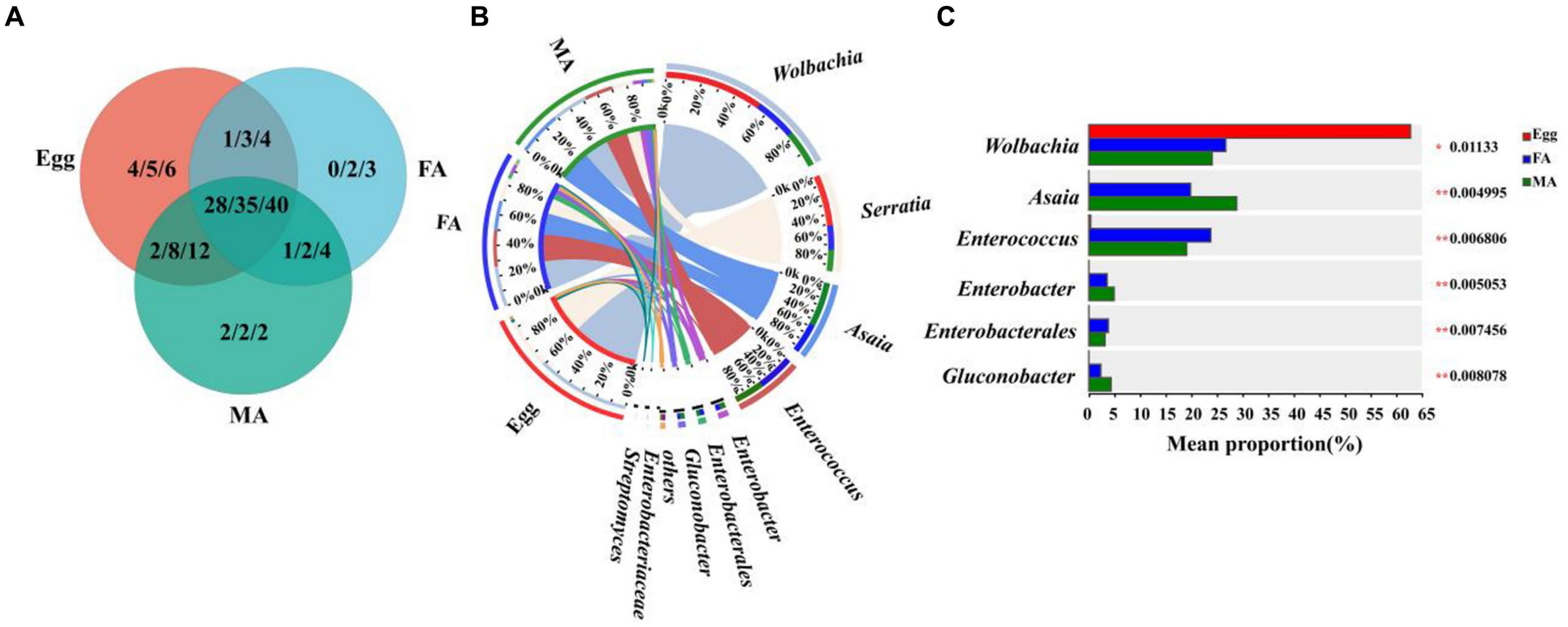
Comparison of symbiont community between *E. balteatus* eggs and adults. **(A)** Shared and unique symbionts between eggs and adults of *E. balteatus* at the level of family, genus, and species. Those three numbers separated by slashes represent the shared and unique symbiont number at family, genus, and species level (from the left to the right), respectively. **(B)** Symbiont composition of *E. balteatus* eggs and female adults (FA) and male adults (MA) at the genus level. **(C)** Top six symbionts with significant differences in relative abundance among eggs and (female and male) adults of *E. balteatus* at the genus level. Egg, FA, MA represent egg, female and male adults of *E. balteatus*, respectively.

To further investigate the potential vertical transmission of symbionts from adults to eggs through reproduction, the microbial communities of adult (female adult, male adult) and egg of hoverfly were analyzed at the family, genus, and species levels, respectively. A variety of symbionts shared by adults and eggs were obtained, belonging to 28 families, 35 genera, and 40 species, respectively (Figure 5A; Supplementary Table S5).

At the genus level, *Wolbachia* was the first dominant symbiont in the three groups (female adults, male adults, eggs), with a relative abundance of 24.08% in male adults, 26.74% in female adults, and 62.80% in eggs (Figures 5B,C; Supplementary Table S5), implying that *Wolbachia* could be vertically transmitted from parents to the eggs after the mating of male and female adults, and they probably played an important role in egg development. The relative abundance of *Serratia,* as the second dominant symbionts, in male and female adults was 13.72 and 15.74%, respectively, and its highest relative abundance in eggs was 33.07% (Figures 5B,C; Supplementary Table S5), indicating that *Serratia* might be also vertically transmitted to the egg from male and female adults. Interestingly, *Asaia*, as the third dominant symbiont, only existed in male and female adults with a relative abundance of 28.86 and 19.87%, respectively, and they did not exist in eggs (Figures 5B,C; Supplementary Table S5), suggesting that it could not be transmitted from adults to eggs through adults vertically.

### 3.5 Effects of diet types on symbiont community of *E. balteatus*

**Figure 6:**
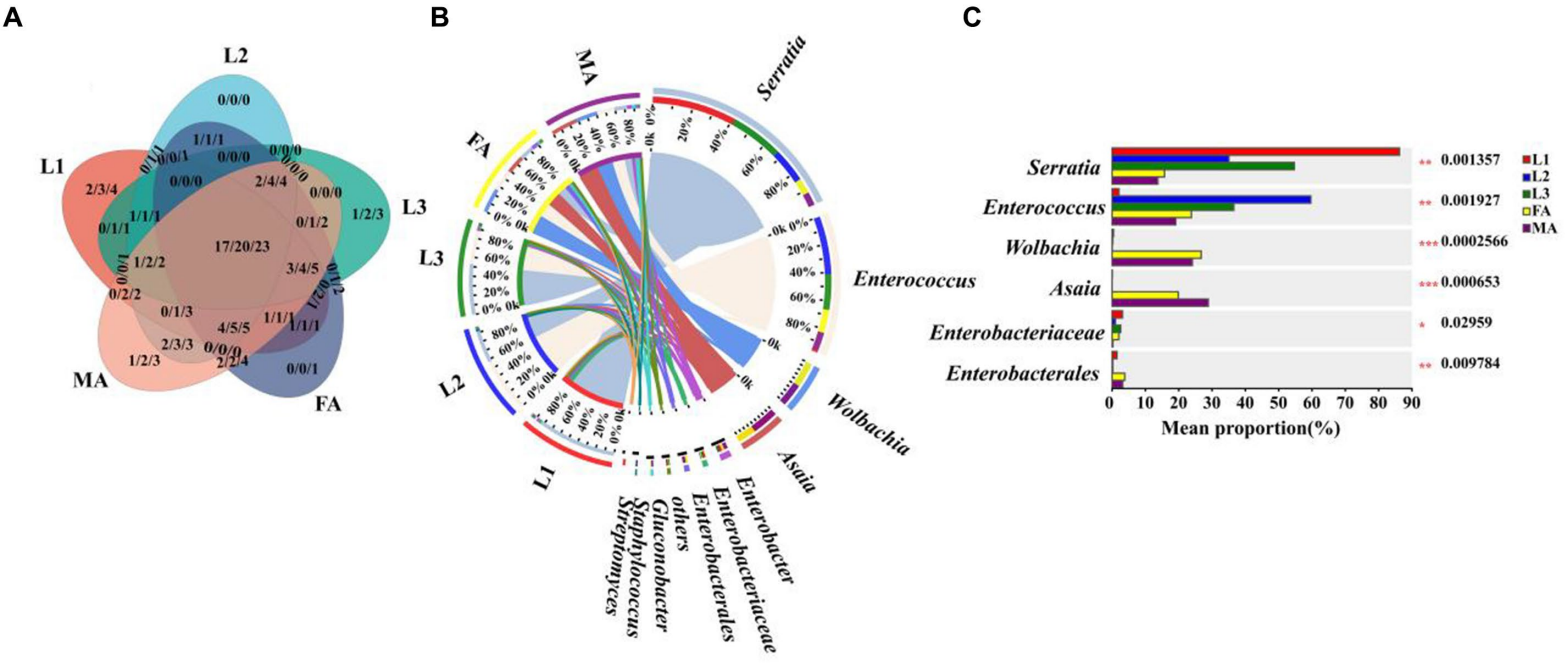
Comparison of symbiont community between *E. balteatus* larvae and adults. **(A)** Shared and unique symbionts between larvae and adults of *E. balteatus* at the level of family, genus, and species level. Those three numbers separated by slashes represent the shared and unique symbiont number at family, genus, and species level (from the left to the right), respectively. **(B)** Composition of symbionts of *E. balteatus* larvae and adults at the genus level. **(C)** Top six symbiont genera with significant differences in relative abundance between larvae and adults of *E. balteatus*. L1, L2, L3, FA, MA represents 1st instar to 3rd instar nymphs, female and male adults of *E. balteatus*, respectively.

To evaluate how diet types influence the symbiont communities of *E. balteatus*, microbiota in *E. balteatus* larvae feeding on aphids and adults feeding on pollens were compared at the family, genus, and species level, respectively. Plenty of symbionts were found shared by larvae and adults of *E. balteatus*, which belonged to 17 families, 20 genera, and 23 species, respectively. Specifically, adult-specific symbiont genera included *Lactobacillus, Bradyrhizobium, Sphingobacterium, Lysobacter*, while larva-specific genera included *Massilia, Empedobacter, Achromobacter, Comamonas, Gammaproteobacteria, Aeromicrobium,* and *Enhydrobacter* (Figure 6A; Supplementary Table S6).

At the genus level, we found that the relative abundance of *Serratia* was significantly higher in larval stages (35.6% ~ 86.24%) than in adult stage (13.72% for male and 15.74% for female) (Figures 6B,C; Supplementary Table S6). Notably, the relative abundance of *Serratia* in aphids was as high as 83.77% (Figure 3C). These results suggested that the *E. balteatus* larvae might obtain *Serratia* bacterium by feeding from aphids, thus maintaining a high *Serratia* relative abundance, whereas the *E. balteatus* adult feeding on pollen could not supplement *Serratia* from aphids, and therefore the relative abundance of *Serratia* decreased. On the contrary, *Asaia* was mainly present in the adult *E. balteatus* with a high relative abundance of 28.86% (for male) and 19.87% (for female), and the relative abundance in *E. balteatus* larvae was extremely low (Figure 6B; Supplementary Table S6). The possible reason might be that the pollen-feeding hoverfly adults could obtain this symbiont from the pollen in comparison to the aphidophagous larvae.

### 3.6 Effects of host sex difference on symbiont community of *E. balteatus*

**Figure 7:**
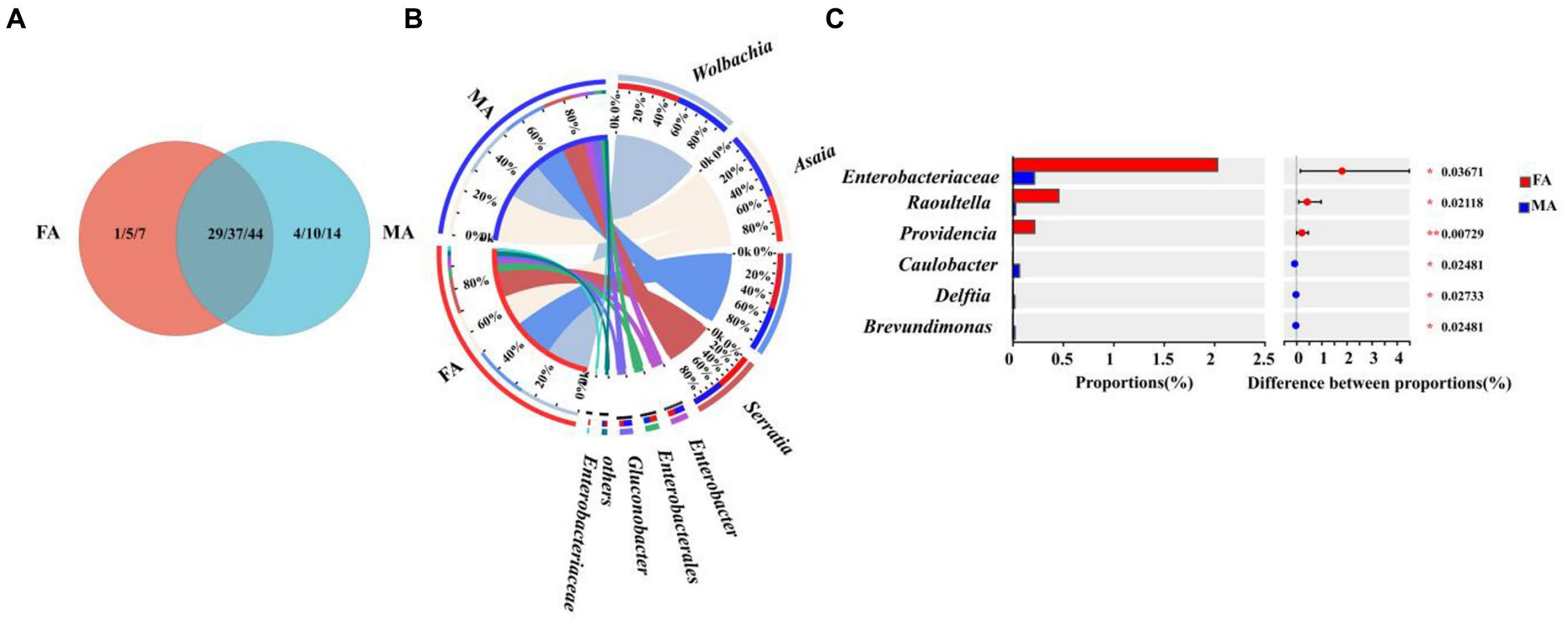
Symbiont community comparison between *E. balteatus* male and female adults. **(A)** Shared and unique symbionts between male and female adults of *E. balteatus* at the family, genus, and species levels. **(B)** Composition of symbionts of *E. balteatus* male and female adults at the genus level. **(C)** Top six symbionts with significant differences in relative abundance between male and female adults of *E. balteatus* at the genus level. FA and MA represent female and male adults of *E. balteatus*, respectively.

To evaluate the effects of host sex difference on the symbiont community of marmalade hoverfly, the symbiont communities in male and female adults of *E. balteatus* were compared at the family, genus, and species levels, respectively. VEEN diagram showed that the symbiont communities shared by of male and female adults were rich, belonging to 29 families, 37 genera, and 44 species (Figure 7A; Supplementary Table S7). At the family, genus, and species level, the number of symbionts unique to male adults was significantly larger than that unique to female adults. Besides, it was found that the *Buchnera* existed only in males, but not in females (Supplementary Table S7).

At the genus level, among the top six symbiont genera in the female and male adults, only *Enterobacteriaceae* had significant differences in relative abundance between the two sex adults, but its relative abundance was very low (2.03% in females and 0.22% in males) (Figures 7B,C; Supplementary Table S7). Taken together, no significant difference in the symbiont composition was observed between male and female adults of *E. balteatus* (Figure 7B).

### 3.7 Biomarker identification across the life cycle of *E. balteatus*

**Figure 8:**
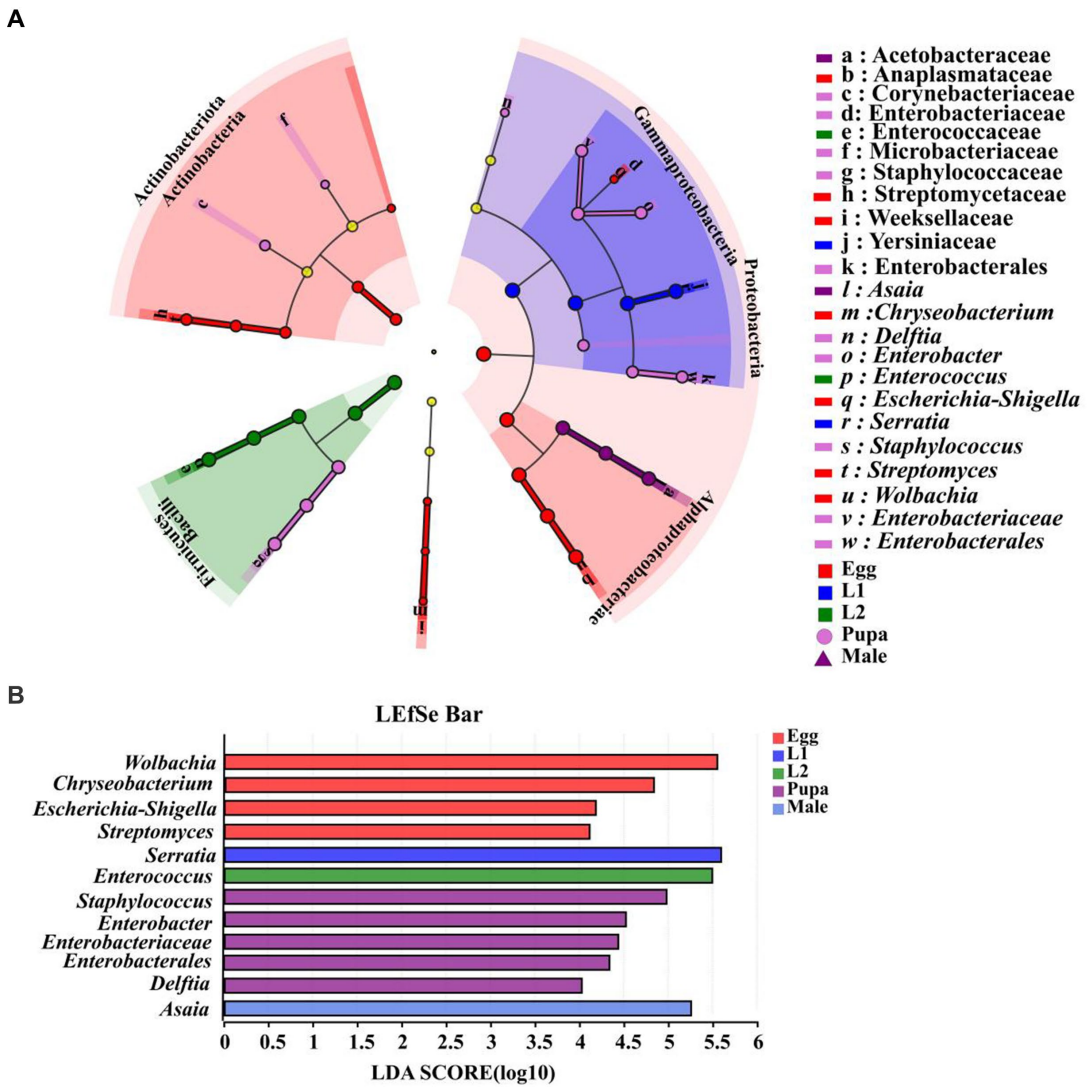
Identification of biomarkers from different developmental stages of *E. balteatus*. **(A)** Biomakers identified from seven samples (eggs, the 1st, 2nd, 3rd instar larvae, pupae, female adults, and male adults) of *E. balteatus* by linear discriminant analysis effect size (LEfSe). In the cladogram, the circles from inside to outside represent the taxonomic level from phylum to genus, and different color nodes represent biomakers in the corresponding taxa. Light yellow nodes indicate biomakers without significant difference among different samples. **(B)** Biomarkers with LDA (linear discriminant analysis) score > 4. Different colors represent biomarkers from different samples.

We analyzed symbiont differences at phylum and genus level in life cycle of marmalade hoverfly. With LDA score (linear discriminant analysis) > 4 as the threshold, we found that there were no significant biomarker symbionts in both 3rd instar larvae and female adults. At the genus level, we identified 12 biomarkers from eggs, the 1st instar larvae, 2nd instar larvae, pupae, and male adult, of which 4 biomarkers were identified from the eggs, including *Chryseobacteriunm*, *Escherichia-Shigella*, *Streptomyces*, and *Wolbachia*. Only one biomarker was identified from the 1st instar larvae and the 2nd instar larvae, which was *Serratia* and *Enterococcus,* respectively. There were 5 biomarkers identified from pupae, which were *Delftia, Enterobacter, Staphylococcus, Enterobacteriaceae,* and *Enterobacterales*, respectively. There was only one biomarker for male adults, namely, *Asaia* (Figures 8A,B).

### 3.8 Function prediction of symbiont community

**Figure 9:**
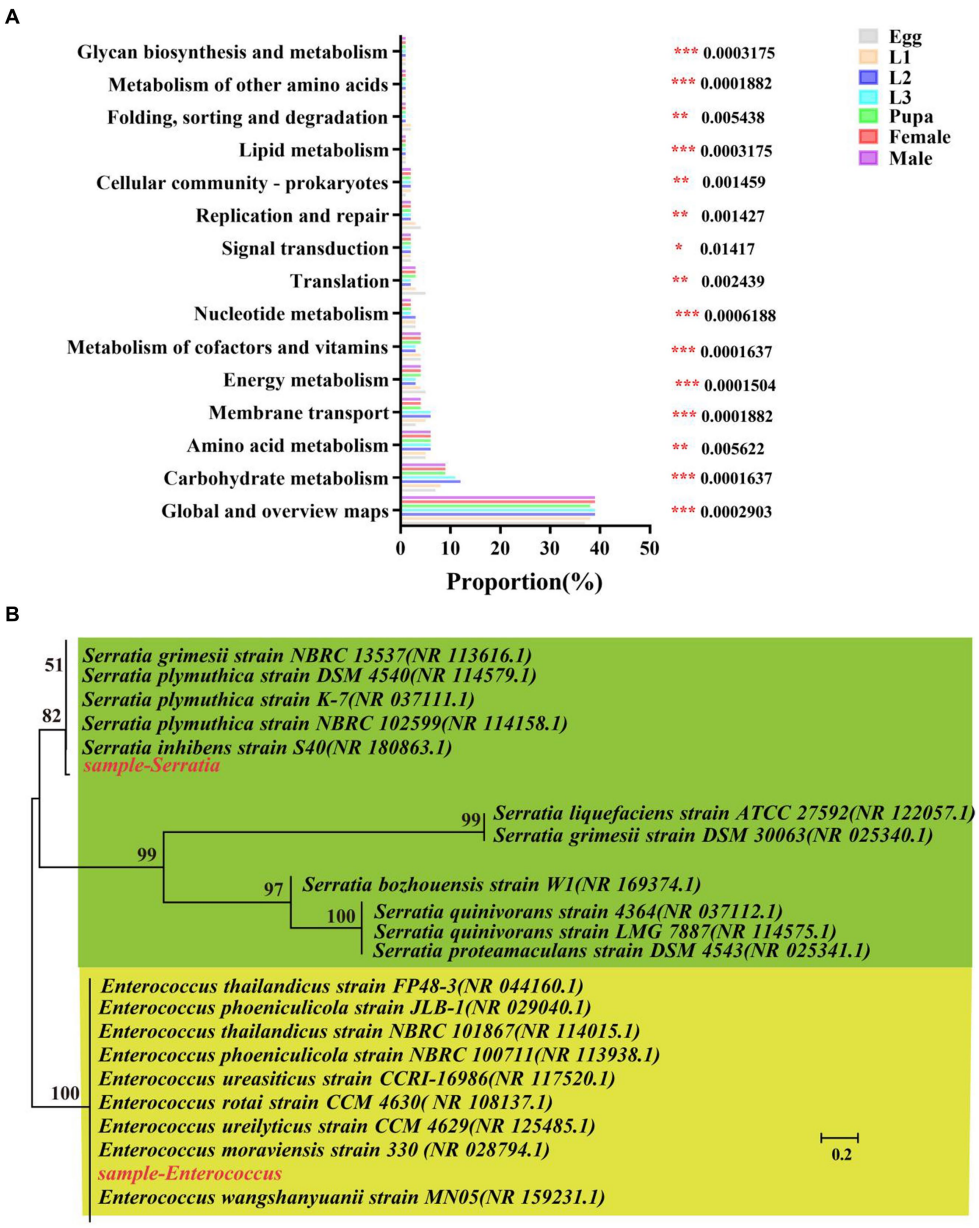
Phylogenetic analysis and functional prediction of symbionts of *E. balteatus* in whole life cycle. **(A)** Functional prediction of symbionts based on 16 s RNA data by KEGG enrichment analysis. The top 15 pathways significantly enriched with high-abundance symbionts are presented on the left. The Tukey–Kramer test was used to determine the statistical significance in high-abundance symbiont enrichment level in the KEGG pathways from the seven samples of hoverfly at 95% confidence interval. *p < 0.05; **p < 0.01; ***p < 0.001. **(B)** Phylogenetic tree of *Serratia* and *Enterococcus.*

Further, we predicted the biological functions of the symbiotic bacteria in hoverfly by PICRUSt2 software and presented top 15 pathways enriched with highest-abundance of symbiotic bacteria (Figure 9A; Supplementary Table S8). These enriched pathways were significantly different across the development stages of the hoverfly. Generally, symbionts with high relative abundance were mainly enriched in 4 pathways including global and overview maps (37–39%), carbohydrate metabolism (7–12%), membrane transport (3–6%), and energy metabolism (3–5%) pathways. Specifically, carbohydrate metabolism was more significantly enriched with symbionts in larval stage higher than in all other stages.

In addition, the phylogenetic tree of *Serratia* and *Enterococcus* was constructed to explore the evolutionary relationship between these two genera (Figure 9B). The phylogenetic tree showed that *Serratia* in the samples was evolutionarily close to *NBRC13537*, but it was distant from *ATCC27592*. The *Enterococcus* in the samples was highly close to *FP48-3*. Furthermore, we also quantitatively verified the relative abundance of these two symbionts (*Serratia* and *Enterococcus*) in all samples (Supplementary Table S1). The absolute abundance of the two dominant symbiotic bacteria was detected by q-PCR method, and it was found that there was a significant difference in the copy number of the two symbiotic bacteria (*p* < 0.001) (Supplementary Figure S3). The results showed that the copy number of *Serratia* was the highest in the first instar larvae, and the copy number of *Enterococcus* was the highest in the second instar larvae. The copy number of the two symbiotic bacteria in the larvae was significantly higher than that in other periods.

## 4 Discussion

Most insects contain symbionts (Oliver and Martinez, 2014), and complex symbiotic relationships between host insects and microorganisms play important roles for both of them. The symbionts are affected by multiple factors such as host food type (Luo et al., 2021; Huang et al., 2022), host gender (Davoodi and Foley, 2020), environment factors (Chen B. S. et al., 2018), and host growth development (Luan et al., 2016).

Our hierarchical clustering analysis revealed that all the samples of *E. balteatus* across the development stages divided into two branches, namely, the “adult & pupa &egg” branch and the larva branch (Figures 1A,B). The bacterial diversity of symbionts in hoverfly larvae was higher than that in hoverfly eggs (Figure 2A), which might be due to the fact that hoverfly larvae fed on aphids. The dominant symbiotic genus in the hoverfly larvae was *Serratia* (Figure 3C). Previous studies have shown that *Serratia* has a certain wax degradation function (Salunkhe et al., 2013; Arunkumar et al., 2022). Another study also found *Serratia,* with genes related to chitin degradation, more abundant in *Novius. pumilus* than in pray *Icerya aegyptiaca*, which may assist their hosts in digesting the wax shell covering the scale insects (Tang et al., 2024). Based on these findings, it could be speculated that *Serratia* may contain chitin degradation-related genes, thus helping their hosts hoverflies digest wax shells covering the aphids, which highlighted the important role of *Serratia* in digesting aphids by predator larvae. Several studies have reported that *Serratia* can maintain and promote the host growth and development by facilitating the metabolisms of amino acids, fatty acids, carbohydrates (Moret et al., 2001; Zhou et al., 2021). Therefore, *Serratia* in *E. balteatus* larvae might mainly exert metabolic and digestive functions. In this study, we found that dominant symbiotic bacteria in hoverfly larval stage were more significantly enriched in the carbohydrate metabolic pathway than in other stages (Figure 9A), indicating a higher nutritional demands and more active metabolism in hoverfly larval stage. Similar results have also been reported in previous study of other dipteran insects (Conceiçao et al., 2021).

In this study, the species richness and community diversity of symbionts in hoverfly from larva to adult stages showed a trend of first increase and then decrease (Figure 2B). This might be due to the change in symbionts in the process of hoverfly eclosion. The intestinal cells of insects can secrete a peritrophic matrix to wrap the intestine contents and the symbiotic bacteria, but during the eclosion and molting process of the complete metamorphosis insects, each organ is remodeled, thus leading to the change in the intestinal symbiotic bacteria homeostasis (Engel and Moran, 2013). Consistently, the community composition of the symbiotic bacteria was significantly different between the marmalade hoverfly adult stage and larva stage (Figures 3C, 6B). Our finding is further supported by one previous report that metamorphosis leads to complete or nearly complete elimination of intestinal bacteria in mosquitoes, and the newly emerging adult intestine contains no bacteria (Moll et al., 2001). These findings jointly suggest that the metamorphosis process is an important factor leading to changes in symbiotic bacteria in insects.

Interestingly, the main symbiotic bacteria genus in the marmalade hoverfly larvae was *Serratia*. One previous study has shown that *Serratia* is a secondary symbiotic bacterium in aphids (Frago et al., 2017). In addition, we also found that the relative abundance of *Serratia* in aphids (83.77%) was similar to that in first-instar larvae (86.24%). Based on these findings, we speculated that the hoverfly could acquire the vast majority of *Serratia* from prey aphids by feeding, which was known as horizontal transmission. This speculation was also confirmed by one previous report that the composition of symbionts in predator ladybirds was highly similar to that in prey aphids (Huang et al., 2022), indicating that symbionts in aphids can be transmitted horizontally to ladybirds.

Furthermore, the main symbionts of marmalade hoverfly adults were *Wolbachia, Asaia*, and *Enterococcus*. *Asaia* has been reported to be a symbiotic bacterium present in plant pollen, and it can enter and colonize the insect intestine with the feeding of insects and can be vertically transmitted in host insects (Favia et al., 2007; Crotti et al., 2009). This is partially in line with our results that *Asaia* only existed in the hoverfly adults feeding on pollen, with its relative abundance in female and male adults being 19.87 and 28.86%, respectively, suggesting that hoverfly adults acquired *Asaia* by feeding pollen, but we did not find the presence of *Asaia* in hoverfly eggs, suggesting that *Asaia* could not be vertically transmitted, which was different from the reports by Favia et al. (2007) and Crotti et al. (2009).

Lastly, the community composition of symbiotic bacteria was highly similar between male and female adults of marmalade hoverfly (Figures 3C, 5B; Supplementary Table S5), indicating that gender was not the main factor affecting the community composition of marmalade hoverfly. *Wolbachia,* as a kind of endosymbiont, has been widely investigated in insect sex research, and it is mainly transmitted vertically, presenting strict maternal transmission (Ahmed et al., 2016). Consistently, we also found its presence in hoverfly eggs, pupae, and adults. *Wolbachia* has been reported to regulate host reproduction and induce feminization, parthenogenesis, and male death of host insects (Bi and Wang, 2020). In this study, we found no difference in the relative abundance of *Wolbachia* between male and female adults.

## 5 Conclusion

This study found that the dominant symbiotic bacteria in the marmalade hoverfly fluctuated across different developmental stages, and the community composition of symbiotic bacteria was not affected. The main symbiotic bacterium in the larval stage is *Serratia*, in the egg and pupal stage is *Wolbachia*, and the adult stage is *Asaia*, respectively. Each developmental stage of hoverfly has its own biomarkers, such as *Serratia* in larval stage and *Asaia* in adult stage. The relative abundance of *Serratia* in the 1st instar larvae of the marmalade hoverfly was close to that in aphids, indicating that marmalade hoverfly obtained these bacteria mainly by feeding on preys. *Asaia* is reported presenting in pollen but dominates in pollen/nectar-feeding marmalade hoverfly adult. Those results both proves that food type is an important factor affecting the difference in symbiotic bacteria between larvae and adults. In addition, we also found that *Wolbachia* mainly exists in the adults and eggs of hoverfly, suggesting that it may be vertically transmitted between generations through host reproduction. Overall, our results demonstrate that both the development process and diet type can affect the symbiotic bacteria dynamics in marmalade hoverfly other than genders, which lays a foundation for further study on the transmission pathway of symbiotic bacteria between hoverfly and food, as well as the colonization and adaptation mechanism of symbiotic bacteria in insects.

## Data Availability

The original contributions presented in the study are publicly available. This data can be found here: https://www.ncbi.nlm.nih.gov/sra, accession number PRJNA1109813.
