# Supplementary material for: Effects of developmental stages, sex difference, and diet types of the host marmalade hoverfly (Episyrphus balteatus) on symbiotic bacteria
Source: Front Microbiol. 2024 Sep 4;15:1433909. doi: 10.3389/fmicb.2024.1433909 (PMC11408942; doi:10.3389/fmicb.2024.1433909)
Supplement: Supplementary file 3 [file Table_3.DOCX]

**Fig. S3 Analysis of 16S rRNA gene copy number of dominant symbiotic bacteria in different samples.**


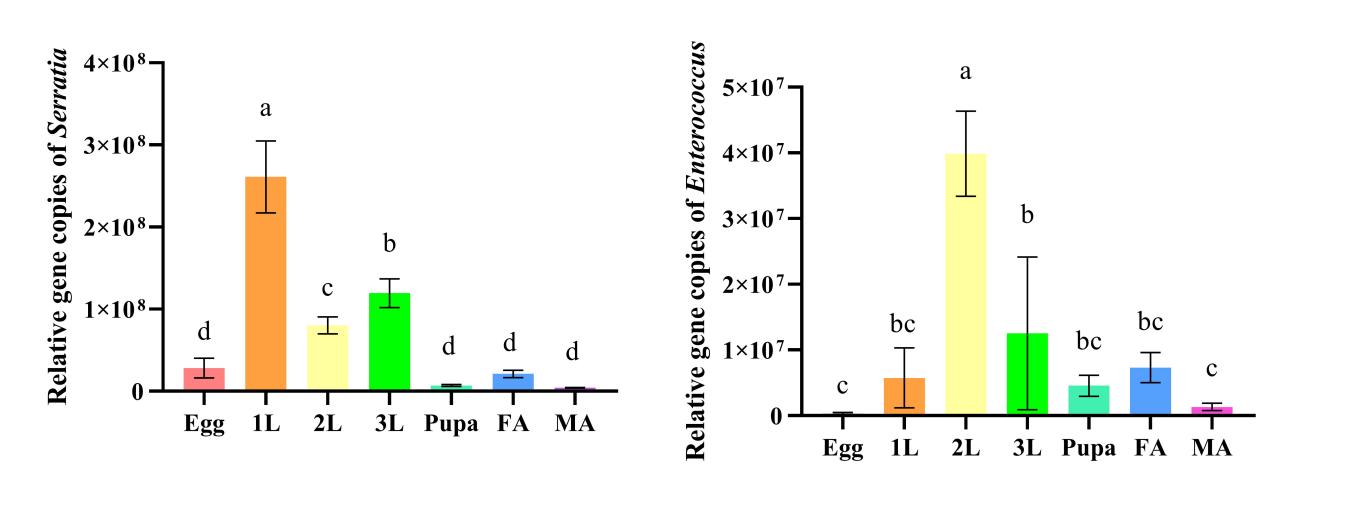


Egg, 1L, 2L, 3L, Pupa, FA, MA represent egg, 1^st^ instar to 3^rd^ instar nymphs, pupa, female and male adults of *E. balteatus*, respectively.
